# Microglia activation in a pediatric rabbit model of tuberculous meningitis

**DOI:** 10.1242/dmm.027326

**Published:** 2016-12-01

**Authors:** Elizabeth W. Tucker, Supriya Pokkali, Zhi Zhang, Vincent P. DeMarco, Mariah Klunk, Elizabeth S. Smith, Alvaro A. Ordonez, Marie-France Penet, Zaver Bhujwalla, Sanjay K. Jain, Sujatha Kannan

**Affiliations:** 1 Department of Anesthesiology and Critical Care Medicine, Division of Pediatric Anesthesiology and Critical Care Medicine, Johns Hopkins University School of Medicine, Baltimore, MD 21287, USA; 2 Center for Infection and Inflammation Imaging Research, Johns Hopkins University School of Medicine, Baltimore, MD 21287, USA; 3 Center for Tuberculosis Research, Johns Hopkins University School of Medicine, Baltimore, MD 21287, USA; 4 Center for Nanomedicine, Johns Hopkins University School of Medicine, Baltimore, MD 21287, USA; 5 Department of Anesthesiology and Critical Care Medicine, Division of Critical Care Medicine, Johns Hopkins All Children's Hospital, St. Petersburg, FL 33701, USA; 6 Department of Pediatrics, Division of Infectious Diseases, Johns Hopkins University School of Medicine, Baltimore, MD 21287, USA; 7 JHU ICMIC Program, Division of Cancer Imaging Research, The Russell H. Morgan Department of Radiology and Radiological Science, Johns Hopkins University School of Medicine, Baltimore, MD 21287, USA; 8 Sidney Kimmel Comprehensive Cancer Center, Johns Hopkins University School of Medicine, Baltimore, MD 21287, USA

**Keywords:** Tuberculosis, Pediatric, Microglia, PET, Meningitis, TSPO

## Abstract

Central nervous system (CNS) tuberculosis (TB) is the most severe form of extra-pulmonary TB and disproportionately affects young children where the developing brain has a unique host response. New Zealand white rabbits were infected with *Mycobacterium tuberculosis* via subarachnoid inoculation at postnatal day 4-8 and evaluated until 4-6 weeks post-infection. Control and infected rabbit kits were assessed for the development of neurological deficits, bacterial burden, and postmortem microbiologic and pathologic changes. The presence of meningitis and tuberculomas was demonstrated histologically and by *in vivo* magnetic resonance imaging (MRI). The extent of microglial activation was quantified by *in vitro* immunohistochemistry as well as non-invasive *in vivo* imaging of activated microglia/macrophages with positron emission tomography (PET). Subarachnoid infection induced characteristic leptomeningeal and perivascular inflammation and TB lesions with central necrosis, a cellular rim and numerous bacilli on pathologic examination. Meningeal and rim enhancement was visible on MRI. An intense microglial activation was noted in *M. tuberculosis*-infected animals in the white matter and around the TB lesions, as evidenced by a significant increase in uptake of the tracer ^124^I-DPA-713, which is specific for activated microglia/macrophages, and confirmed by quantification of Iba-1 immunohistochemistry. Neurobehavioral analyses demonstrated signs similar to those noted in children with delayed maturation and development of neurological deficits resulting in significantly worse composite behavior scores in *M. tuberculosis*-infected animals. We have established a rabbit model that mimics features of TB meningitis in young children. This model could provide a platform for evaluating novel therapies, including host-directed therapies, against TB meningitis relevant to a young child's developing brain.

## Abstract (precis)

**Summary:** A clinically relevant novel rabbit model provides robust microglial activation and neurological deficits as seen in children with pediatric central nervous system tuberculosis.

## INTRODUCTION

Globally, central nervous system (CNS) tuberculosis (TB) continues to be a devastating disease that disproportionately affects toddler-age children (Be et al., 2009; Jain et al., 2013; Yaramis et al., 1998). CNS TB most commonly presents as meningitis, but can also present as intracranial tuberculomas or, less frequently, tuberculous brain abscesses (Be et al., 2009; Jain et al., 2005; Kumar et al., 2002; Rock et al., 2008). TB meningitis is the most severe form of TB and is associated with significant morbidity and mortality (13-57%) even with the completion of twelve months of arduous treatment (Girgis et al., 1998; Rohlwink et al., 2016a; van Well et al., 2009; Yaramis et al., 1998). Children often present with signs of increased intracranial pressure, brainstem dysfunction, cranial nerve palsies and stroke resulting in permanent hemiplegia and quadriplegia (Jain et al., 2013; van Well et al., 2009; Yaramis et al., 1998). Neuroinflammation seems to be a key component of the pathological process, leading to exudative meningitis, endovasculitis, infarction and obstructive hydrocephalus seen as meningeal or post-contrast enhancement on computed tomography (CT) or magnetic resonance imaging (MRI), associated with neurologic disability and poor outcomes (Be et al., 2009; Donald and Schoeman, 2004; Katti, 2004; Schoeman and Donald, 2013; van Well et al., 2009). Host-directed therapies have been used for the treatment of TB meningitis with strong evidence for the benefit of adjunctive corticosteroids in decreasing mortality in adults (Prasad and Singh, 2008; Thwaites et al., 2004). Additionally, in children, adjunctive corticosteroids have been shown to not only be beneficial in reducing mortality, but also in reducing neurologic sequelae (Girgis et al., 1991; Schoeman et al., 1997). However, corticosteroid use leads to a nonspecific modulation of the immune response with significant side effects that can limit its use (Ordonez et al., 2014). Therefore, there is an urgent need for the development and validation of novel host-directed therapies for CNS TB.

Although animal models of TB meningitis have been previously developed, they typically mimic adult disease (Tsenova et al., 1999, 2005, 2002, 1998; van Well et al., 2007) and do not take into account the effects of injury and immune dysregulation in the developing brain, which would have significant relevance in childhood disease. Microglia, the resident immune cells in the brain, are the primary host cells of *Mycobacterium tuberculosis* and release pro-inflammatory cytokines when activated as a result of TB infection (Curto et al., 2004; Hernandez Pando et al., 2010; Rock et al., 2005; Yang et al., 2007, 2009; Zucchi et al., 2012). Microglia are not only involved with host-defense (Rock et al., 2004), but also have a crucial role in neurodevelopment. During development, microglia are active in axonal guidance, neurodevelopmental apoptosis and synaptogenesis (Schafer et al., 2012; Tremblay et al., 2010; Verney et al., 2010). CNS infection leads to the disruption of normal glial function, thereby making the immature brain uniquely vulnerable to injury.

In this study, we developed a rabbit model that mimics features of TB meningitis in young children. Rabbits were chosen as the progression of myelination, microglial presence in the white matter tracts, and brain development follows a pattern that is similar to that in humans where it starts perinatally and continues in the postnatal period, albeit in a more compressed timeframe (Drobyshevsky et al., 2005; Saadani-Makki et al., 2008). Subarachnoid infection with *M. tuberculosis* led to the development of inflammatory meningitis, characteristic TB lesions and microglial activation as early as two weeks after infection in this model, corresponding to a postnatal age where several neurological functions are still maturing in the rabbit. Neurobehavioral analyses, and non-invasive live animal imaging with MRI and positron emission tomography (PET), targeting activated microglia/macrophages (Foss et al., 2013; Ordonez et al., 2015), were correlated with CNS disease as seen on postmortem examination.

## RESULTS

### Establishment of CNS infection

**Fig. 1.**
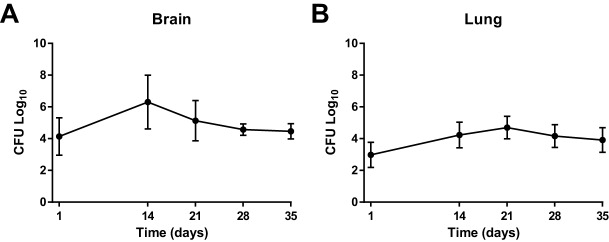
**Bacterial growth kinetics in the brain and lung.** Colony forming units (CFU) demonstrate exponential bacillary growth after injection in the brain. (A) A greater bacterial burden was seen in the brain, as anticipated. Day 1 brain implantation mean±s.d. was 4.14±1.18 log_10_. Peak bacterial growth was noted to be at 14 days post-injection in the brain (mean±s.d.=6.31±1.7 log_10_) and stabilized by 28 and 35 days post-infection (mean±s.d.=4.57±0.36 log_10_ and mean±s.d.=4.46±0.49 log_10_, respectively). (B) Day 1 lung implantation mean was 2.97±0.79 log_10_ with an increase at 14 days post-infection (mean±s.d.=4.23±0.81 log_10_) and peak bacterial growth at 21 days (mean±s.d.=4.69±0.71 log_10_). Bacillary load decreased and stabilized by 28 and 35 days post-infection (mean±s.d.=4.16±0.72 log_10_ and mean±s.d.=3.91±0.78 log_10_, respectively). Lung bacterial growth indicates that dissemination of the bacteria occurs from the brain to the bloodstream and other organs in the body. Data represents at least four animals for each time point (day 1, *n*=4; day 14, *n*=4; day 21, *n*=11; day 28, *n*=7; day 35, *n*=5).

We performed direct inoculation of *M. tuberculosis* in post-natal day (PND) 4-8 rabbits via subarachnoid route to produce CNS infection. Mean bacillary implantation (one day after subarachnoid infection) was 4.14±1.18 log_10_ colony forming units (CFU) (mean±s.d.) and reached 6.31±1.7 log_10_ 14 days post-infection (Fig. 1A). The bacterial burden remained relatively stable thereafter. Further, we observed bacterial spread from brain to lungs, which might be a result of disruption of the blood-brain barrier (BBB) (Fig. 1B), suggesting that like young children, young rabbits have limited capacity to prevent dissemination of infection.

### TB lesions and inflammatory exudate

**Fig. 2.**
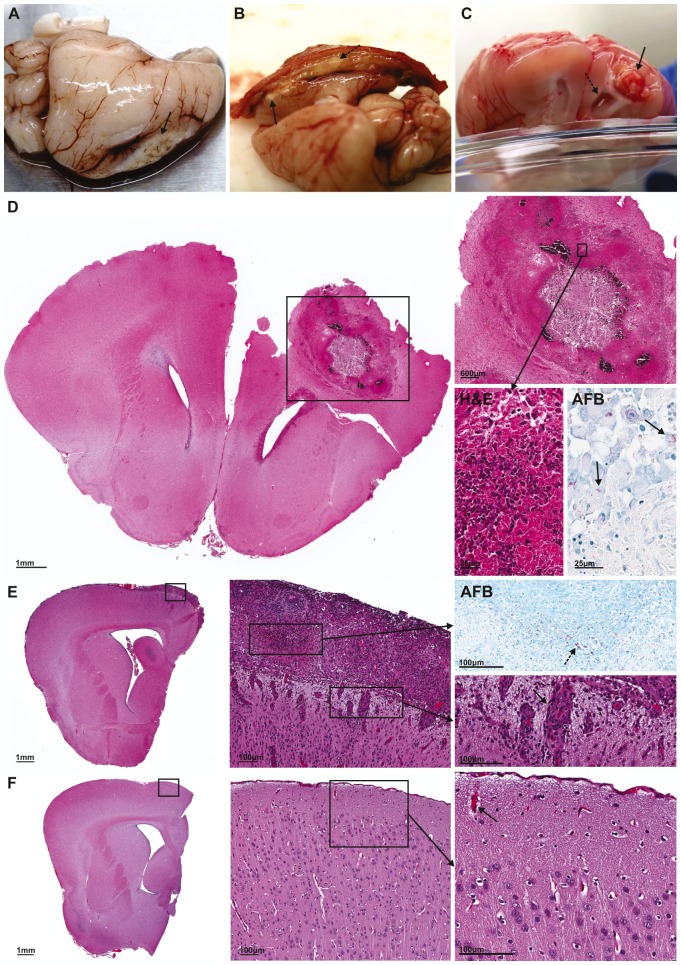
**Representative gross pathological and microscopic examination of brain lesions.** (A) Superficial, medial tuberculoma formed at 14 days post-infection. (B) Medial tuberculoma and exudate (dashed arrow) adhering brain to skull at 21 days post-infection. (C) Deep tuberculoma associated with hydrocephalus (dashed arrow) formed bilaterally but greater on side ipsilateral to tuberculoma at 35 days post-infection. Tuberculomas are indicated by small black arrows in panels A-C. (D) Microscopic examination of tuberculoma from panel C demonstrates the localization of the tuberculoma to one hemisphere. High-power view (inset) demonstrates central necrosis and dense cellular rim (H&E), with Ziehl–Neelsen stain (AFB) highlighting the numerous *M. tuberculosis* bacilli in the cellular rim. *M. tuberculosis* bacilli stained as red rods (black arrows). (E) Microscopic examination of one hemisphere of *M. tuberculosis*-infected brain with inflammatory meningitis. High-power view (inset) highlights numerous red *M. tuberculosis* bacilli (dashed arrow) with Ziehl–Neelsen stain (AFB) and demonstrates perivascular infiltrate (black arrow) associated with the inflammatory exudate (H&E). (F) Microscopic examination of one hemisphere of control brain. High-power view (inset) demonstrates no inflammatory exudate and normal vessels (black arrow) with no perivascular infiltrate.

We observed a diffuse inflammatory response with exudative meningitis, causing adhesion of the brain parenchyma to the skull even without localized TB lesion formation (87%, 26/30; Fig. 2B). Inflammation of meninges was associated with perivascular infiltrate (Fig. 2E). Localized infection with tuberculoma formation was observed in most of the infected animals (80%, 24/30) as early as 14 days post-infection (Fig. 2A-D). The majority of the tuberculomas were superficial in the frontotemporal lobe close to the site of injection (Fig. 2A,B). However, one animal developed a tuberculoma deeper in the parenchyma, with the lesion within the ventricle causing notable hydrocephalus (Fig. 2C). By contrast, control animals had no inflammation of the meninges or perivascular inflammatory infiltrate (Fig. 2F). Histopathological examination of the TB lesions demonstrated characteristic TB granulomas with central necrosis and dense cellular rim on H&E staining, and numerous *M. tuberculosis* bacilli around these lesions on Ziehl–Neelsen staining (Fig. 2D).

### Microglial activation is increased in *M. tuberculosis*-infected animals

**Fig. 3.**
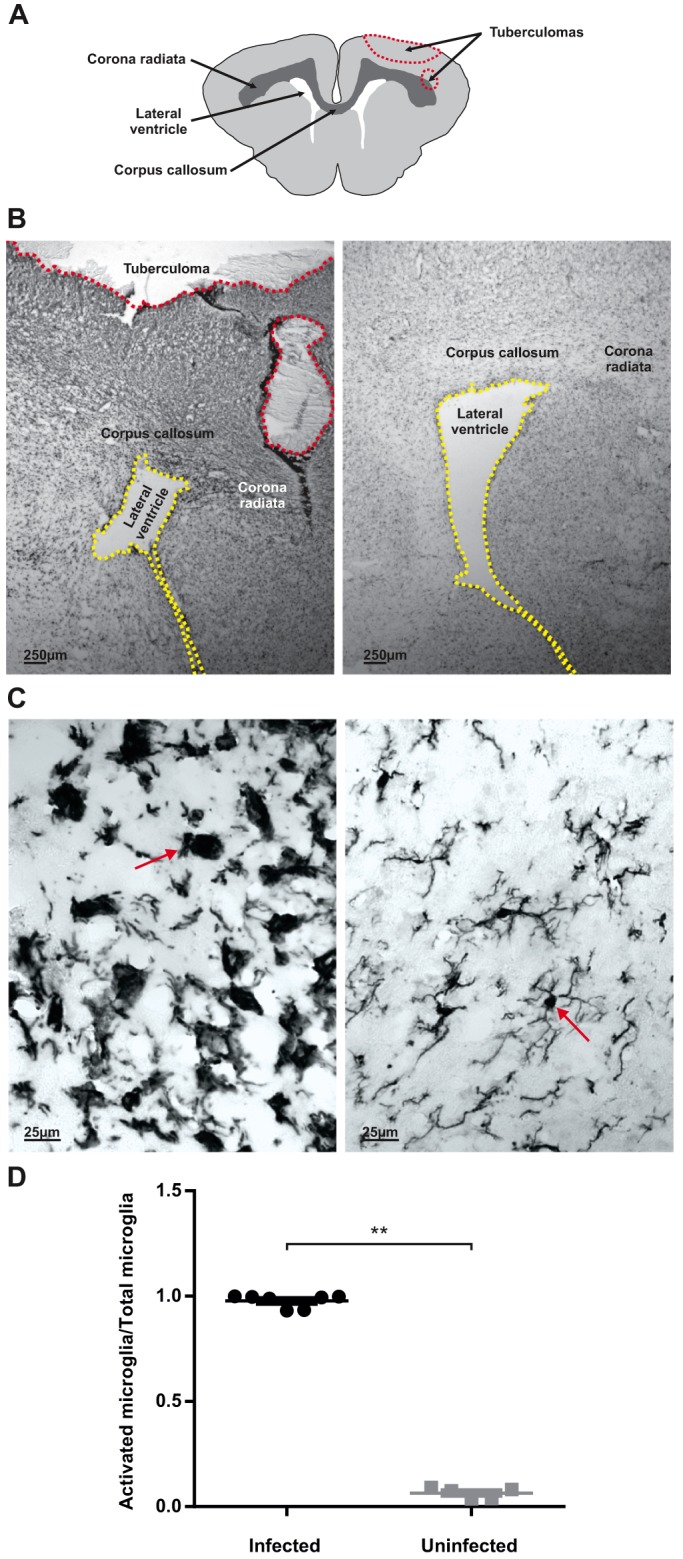
**Microglia are activated in TB meningitis.** (A) Schematic representation of rabbit brain section highlighting lateral ventricles, corpus callosum, corona radiata and tuberculoma (red dashed line) seen at higher magnification in brain tissue slices in B. (B-C) Representative Iba-1-stained brain tissue sections from TB-infected (B,C, left panels) and control (B,C, right panels) animals at 21 days post-infection (PND 3-4 weeks) shown under low (B) and high (C) magnification. The low-magnification images (B) show the lateral ventricles (yellow dashed line), corpus callosum, corona radiata and tuberculoma (red dashed line). The high-magnification images (C) are from the region of the corpus callosum. *M. tuberculosis*-infected animals (B,C, left panels) demonstrates dense, activated microglia with large cell bodies and shortened and/or thickened processes (red arrow) whereas microglia in age-matched controls demonstrate a ‘normal’, ‘resting’ morphology with long, thin processes and small cell bodies (red arrow). (D) Quantitative analyses in the corpus callosum demonstrates a significant increase in percentage of activated microglia in *M. tuberculosis*-infected rabbit kits compared with controls. *M. tuberculosis*-infected mean±s.e.m.=0.98±0.01, *n*=7; control mean±s.e.m.=0.06±0.01, *n*=5; unpaired *t*-test, **P≤0.01.

The expression of microglia/macrophage-specific calcium-binding protein, ionized calcium-binding adapter molecule 1 (Iba-1, also known as Aif1) was used to assess microglial presence and activation. Brain tissue sections from control animals showed ‘inactivated’ or ‘resting’ microglia characterized by long, thin cell processes and small cell bodies. However, brain tissue from *M. tuberculosis*-infected animals demonstrated intense ‘activated’ microglia with morphologic changes, indicated by large cell bodies and thick, short processes (Fig. 3B,C). All images were quantitatively analyzed for the ratio of activated microglia to total microglia cells (activation index) in the white matter (corpus callosum). A statistically significant increase in the ratio of activated microglia was observed in brain tissue of *M. tuberculosis*-infected (activation index mean±s.e.m.=0.98±0.01) versus control animals (activation index mean±s.e.m.=0.06±0.01; *P*<0.01 for infected versus control; Fig. 3D). When tuberculomas were present, there was an increased presence of Iba-1 around TB lesions where the bacilli localized, signifying a high density of activated microglial morphology in these regions, whereas the microglial cells farther from the infection foci were more ramified with longer processes and smaller cell bodies (Fig. S2).

### ^124^I-DPA-713 PET/CT imaging confirms the presence of activated microglia/macrophages localized to the TB lesion

**Fig. 4.**
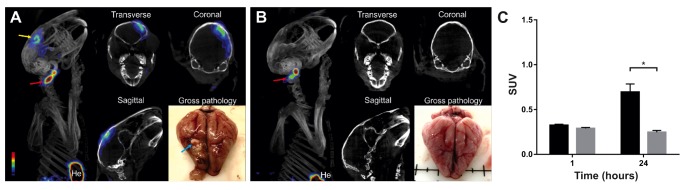
**^124^I-DPA-713 PET/CT imaging demonstrates tracer uptake localizing to the TB lesion.** (A) Three-dimensional reconstruction, transverse, coronal and sagittal views of co-registered PET/CT images and gross pathology from representative *M. tuberculosis*-infected animal at 21 days post-infection and 24 h post-tracer injection. Gross pathology shows a discrete TB lesion in right hemisphere (blue arrow), and PET signal localizes to the TB lesion (yellow arrow) and the site of injection (ear vein, red arrow). (B) Three-dimensional reconstruction, transverse, coronal and sagittal views of co-registered PET/CT images and gross pathology from representative control animals at same time point. Gross pathology shows no lesions in the control animal, with minimal PET signal at the site of injection (red arrow). (C) Quantification of the PET signal demonstrates significant higher activity in *M. tuberculosis*-infected versus controls animals at 24 h post-tracer injection. *M. tuberculosis*-infected SUV mean±s.e.m.=0.70±0.09, *n*=4; control SUV mean±s.e.m.=0.25±0.02, *n*=2; unpaired *t*-test, *P=0.03. He, heart.

Co-registered PET/CT images from representative *M. tuberculosis*-infected and control animals are shown in Fig. 4. PET signal was localized to the TB lesion (corresponding to the location noted on postmortem gross pathological examination) in *M. tuberculosis*-infected brain compared with the control animals at 24 h post-tracer injection [*M. tuberculosis*-infected standardized uptake value (SUV) mean±s.e.m.=0.70±0.09, control SUV mean±s.e.m.=0.25±0.02; *P*=0.03; Fig. 4C]. 3D reconstruction videos are available (Movies 1 and 2).

### MR imaging demonstrates gadolinium enhancement of TB lesions

**Fig. 5.**
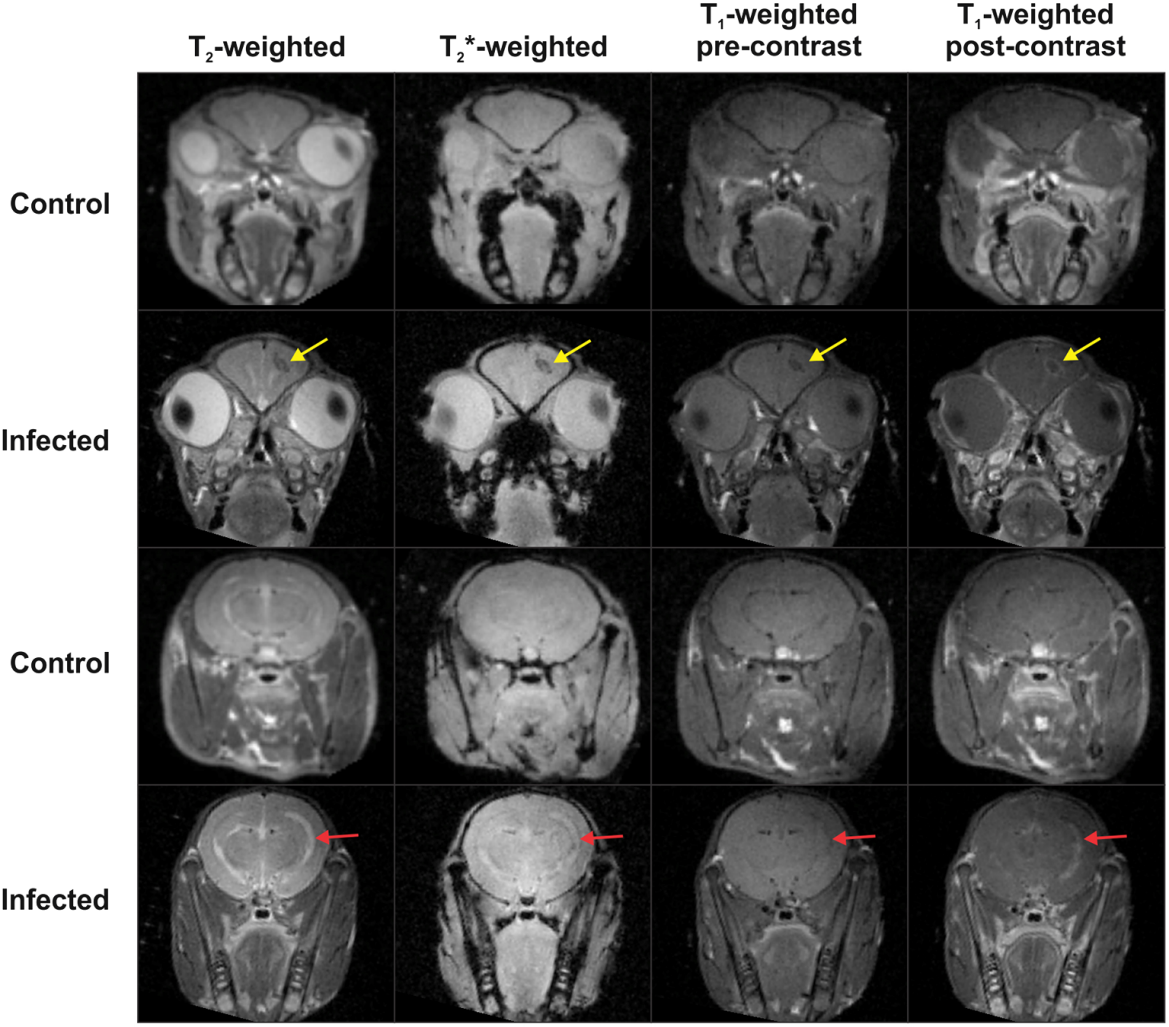
**MR imaging demonstrates gadolinium enhancement of TB lesions.** Representative coronal MR images of 1 mm thick slices of control and *M. tuberculosis*-infected animals at the site of injection (rows 1 and 2) and posterior to site of injection (rows 3 and 4) at 28 days post-infection. Control animal in first and third row demonstrates normal MRI with T_2_-, T_2_*-, pre- and post-contrast T_1_- weighted images. Row 2 shows hypodense TB lesion in right hemisphere with post-contrast T_1_-weighted enhancement of the lesion (yellow arrow) in the *M. tuberculosis*-infected animal. Row 4 demonstrates enlarged ventricle in the T_2_-weighted image with visible post-contrast enhancement in the T_1_-weighted image (red arrow) in the *M. tuberculosis*-infected animal.

Representative MR imaging of *M. tuberculosis*-infected and control animals 28 days post-infection are shown in Fig. 5. Imaging of *M. tuberculosis*-infected animals showed post-gadolinium enhancement of the tuberculomas on T_1_-weighted images and T_2_-weighted images demonstrated enlarged ventricles with enhancement ipsilateral to the tuberculoma. The post-gadolinium enhancement of TB lesions seen in this model is similar to enhancement of tuberculomas in individuals with CNS TB (Katti, 2004; Rohlwink et al., 2016b).

### Neurobehavioral testing shows decreased maturity and loss of function and/or motor deficits in *M. tuberculosis*-infected animals

**Fig. 6.**
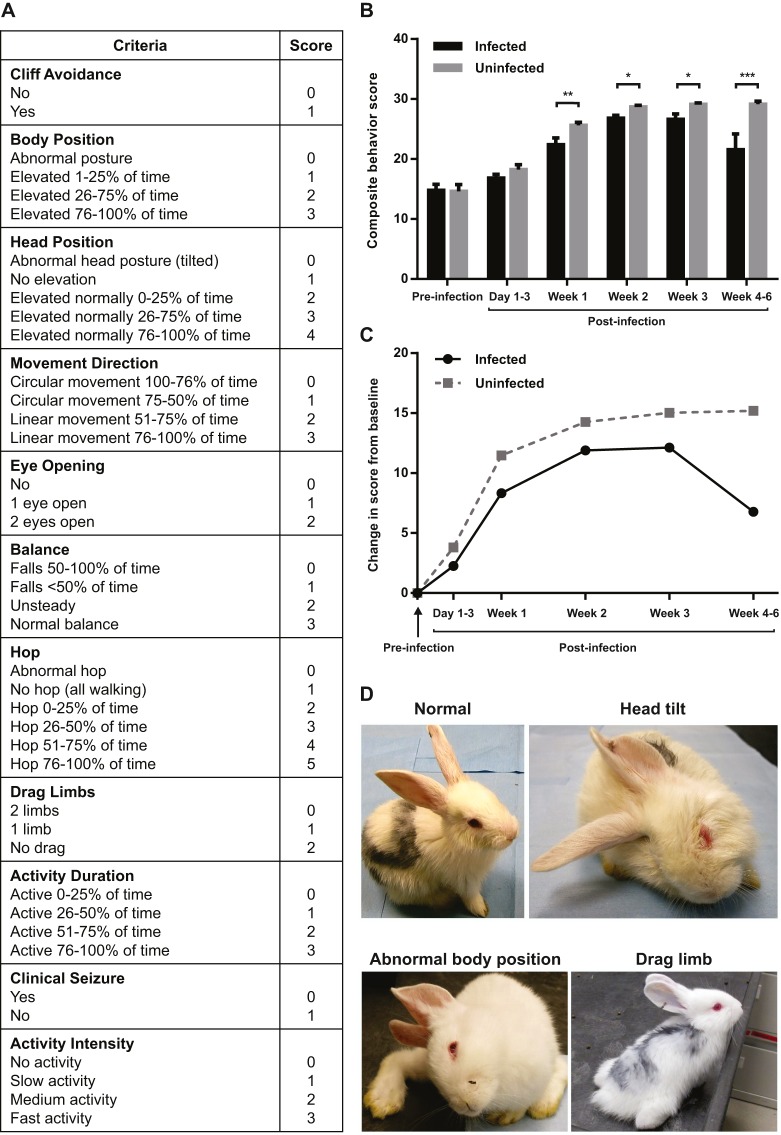
**Neurobehavioral testing shows decreased maturity and loss of function/motor deficits in *M. tuberculosis*-infected animals.** (A) Composite behavior score criteria. Lower scores reflect decreased maturity and/or loss of function/motor deficits. (B) Composite behavior scores were significantly lower in *M. tuberculosis*-infected animals at 1 week (*M. tuberculosis*-infected 22.38±1.14, *n*=20; control 25.61±0.52, *n*=14; *P*=0.01), 2 weeks (*M. tuberculosis*-infected 26.82±0.47, *n*=28; control 28.69±0.26, *n*=18; *P*=0.03), 3 weeks (*M. tuberculosis*-infected 26.63±0.88, *n*=20; control 29.11±0.24, *n*=14; *P*=0.02) and 4-6 weeks (*M. tuberculosis*-infected 21.55±2.63, *n*=7; control 29.17±0.5, *n*=4; *P*<0.01) post-infection compared with controls. *P<0.05; **P≤0.01; ***P≤0.001. Composite behavior scores are presented as mean±s.e.m. and statistical analysis was done by linear mixed models. (C) Maturation assessment comparing change in composite behavior score from baseline over time. This graph of the change in behavior from baseline at each time point demonstrates the delayed rate of achievement of milestones in the *M. tuberculosis*-infected animals and subsequent loss of function when compared with controls. The level of maturation achieved by control animals at postnatal age of ∼14 days (corresponds to 1 week post-infection) was seen only at 3 weeks of age (2 weeks post-infection), in the *M. tuberculosis*-infected animals. (D) Left, upper picture is a control animal with normal head position and motor control of limbs. Right, upper picture is *M. tuberculosis*-infected animal with head tilt to right. Left, lower picture is *M. tuberculosis*-infected animal with head tilted to right, inability to hop, and abnormal body position. Right, lower picture is a *M. tuberculosis*-infected animal with limb dragging.

Neurobehavioral scores were compared between the control and *M. tuberculosis*-infected groups over time. At time 0 (pre-infection) there were no differences between the groups. However, neuro-monitoring over time demonstrated that the *M. tuberculosis*-infected animals developed clinically apparent neurological deficits as early as 2 weeks post-infection, with abnormal gait, seizures, head tilt and motor deficits of the hind limbs (Fig. 6D). Representative videos are shown in Movie 3. The composite behavior score reflects both delays in normal development and progressive loss of function. Newborn animals are normally not able to hop and have their eyes closed and their motor function improves as they mature. However, in the presence of infection there is delayed achievement of normal milestones such as eye opening, and loss of motor functions. As the infection progressed, animals began to drag their hind limbs, hopped less frequently or lost normal head and body elevation, leading to a decrease in the scores. A significant decrease in scores was seen for the *M. tuberculosis*-infected animals when compared with the control animals by 7 days post-infection, and this difference worsened over time (Fig. 6B). A delay in maturation was seen in the juvenile rabbits with TB meningitis when compared with the controls (Fig. 6C). We observed a variation in phenotypic severity of the *M. tuberculosis*-infected animals, with severe signs developing in 23% (6/26) animals that developed meningitis and TB lesions on gross pathologic examination.

## DISCUSSION

The burden of TB in children remains high, and the World Health Organization (WHO) estimates 1 million new cases of TB in children annually (World Health Organization, 2016). This is a substantial increase from the prior WHO (2014) estimation, and represents the challenges of diagnosing TB in children, which is rarely bacteriologically confirmed (Salazar-Austin et al., 2015). TB meningitis continues to be associated with high morbidity and mortality in young children. Therefore, the development of relevant preclinical models is urgently needed to facilitate research investigating its pathogenesis and develop new treatments and tools to better diagnose and monitor diseases.

Seminal animal studies performed by Rich and McCordock (1933) reported that TB meningitis resulted from the rupture of ‘rich foci’ into the subarachnoid space, which developed around bacilli deposited in brain parenchyma much earlier during hematogenous spread. Several models have elucidated key pathogenic pathways and bacterial factors involved in neuropathogenesis (Be et al., 2008; Hernandez Pando et al., 2010; Jain et al., 2006; Rich and McCordock, 1933; Tsenova et al., 1998; Yang et al., 2007; Zucchi et al., 2012). For example, an *in vitro* model of the BBB identified *M. tuberculosis* microbial factors associated with neuro-invasiveness (Jain et al., 2006). Mouse and guinea pig models of CNS TB, developed by Be et al. (2011) and Hernandez Pando et al. (2010), suggested that *M. tuberculosis* has strain-specific CNS dissemination and pathology, reflecting virulence factors that might enable invasion and survival in the CNS.

Many elegant studies have also been performed by using direct intra-cisternal infections in adult mice or rabbits (Tsenova et al., 1999, 2005, 2002, 1998; van Well et al., 2007). Several animal studies have explored host-pathogen interactions by targeting critical inflammatory pathways implicated in TB pathogenesis (Ong et al., 2014; Ordonez et al., 2016a; Skerry et al., 2012), as well as developing novel adjunctive treatments for TB meningitis (Majeed et al., 2016; Tsenova et al., 2002, 1998). However, these animal models do not evaluate the effect of TB meningitis and the host immune response on normal brain development, which would be crucial in the immature brain. Some studies suggest that there is a higher prevalence of cerebral infarcts and strokes in children with TB meningitis, which are associated with worse prognosis (Dastur et al., 1970; Kingsley et al., 1987; Rohlwink et al., 2016a,b). Therefore, more clinical and preclinical studies are needed to evaluate the potentially different host immune response in the younger and more immature brain.

*In vitro* studies by Curto et al. (2004) and Rock et al. (2005) established microglia cells as the primary host cells of *M. tuberculosis*, causing activation and release of pro-inflammatory cytokines such as tumor necrosis factor-α (TNF-α). Both adult animal models and post-mortem human brain biopsies have also shown that activated microglia are recruited to TB lesions (Green et al., 2011; Hernandez Pando et al., 2010; Zucchi et al., 2012), and are noted to have intracellular bacilli (Hernandez Pando et al., 2010). Microglia also play a crucial role in the young brain during normal development. Microglia are essential for axonal and synaptic plasticity through axonal guidance, neurodevelopmental apoptosis, neurogenesis and synaptogenesis, making them vital for normal development (Cunningham et al., 2013; Schafer et al., 2012; Tremblay et al., 2010; Verney et al., 2010). Therefore, we focused on developing a model that mimics TB meningitis in toddler-age children. The clinical relevance of this model lies in the ability to study the microglial response during active white matter development and myelination mimicking that in children. To date, corticosteroids are the only host-directed therapy proven to decrease mortality and morbidity in children with TB meningitis (Girgis et al., 1991; Prasad and Singh, 2008; Schoeman et al., 1997). Therefore, a better understanding of the role of immune cells in the developing brain involved in TB meningitis is imperative for developing novel therapeutic strategies that would improve outcomes while facilitating normal brain development.

Subarachnoid infection in our model led to inflammatory meningitis and formation of characteristic brain tuberculomas with central necrosis and dense cellular rim in the majority of *M. tuberculosis*-infected animals. Diffuse exudative meningitis and hydrocephalus seen on gross pathological examination, as well as MRI with gadolinium enhancement, akin to human disease, were also noted. Histologically, microglia were robustly activated by *M. tuberculosis* infection, confirming the integral involvement of microglia described previously by Curto et al.’s (2004) and Rock et al.’s (2005) *in vitro* studies and in other animal models by Hernandez Pando et al. (2010) and Zucchi et al. (2012). In order to non-invasively monitor microglial inflammation in our model, we used ^124^I-DPA-713, a newer generation of TSPO ligand, with excellent signal-to-noise ratios for the detection of activated microglia/macrophages associated with *M. tuberculosis* infection (Foss et al., 2013; Ordonez et al., 2015). In our model, ^124^I-DPA-713 PET/CT imaging demonstrated signal localized to the TB lesion, with low background signal in control animals. Detecting microglial activation using non-invasive PET *in vivo* imaging could potentially be used as a biomarker for early diagnosis and treatment monitoring. The extent of microglial activation using PET imaging has been shown to correlate with the severity of neurologic injury in a neonatal rabbit model of cerebral palsy (Kannan et al., 2011, 2007) and might also be helpful as a prognostic indicator. First-in-human studies using ^124^I-DPA-713 are currently ongoing. Pathogen-specific imaging modalities to specifically detect bacteria directly could also substantially enhance the diagnostic and monitoring capabilities (Ordonez et al., 2016b; Weinstein et al., 2014) and could be tested in this model in future studies. MRI could also be utilized to monitor response to treatment or predict outcomes. A recent prospective pediatric study by Rohlwink et al. (2016b) demonstrated that MRI evidence of severe infarcts from either vascular or non-vascular pathology, such as intracranial hypertension or hydrocephalus, was predictive of poor outcomes. Although we did not identify infarcts in our model at the time of imaging, enlarged ventricles were detected on MRI. It is possible that infarcts could be visualized at later time points with disease progression.

There was a wide variability in the neurobehavioral scores correlative of disease severity in *M. tuberculosis*-infected rabbits, with worsening signs as the infection progressed. The differences in composite behavior scores noted at the early time points primarily correlates with delays in normal developmental milestones that are indicative of the early signs of infection in these animals. Retrospective studies in children with TB meningitis found that children were more likely to present with advanced disease (stage II with lethargy, nuchal rigidity, seizures and focal neurological signs often with cranial nerve abnormalities or stage III with hemiplegia or paraplegia, coma and eventual death) and only 3-10% present with mild disease (stage I with fever, headache and loss of developmental milestones) (Long et al., 2012; van Well et al., 2009; Yaramis et al., 1998). Our animal model could enable further characterization of pediatric TB meningitis over time to better delineate the pathogenesis when the majority of children present with advanced disease.

The current study has some limitations. The developmental time frame is much more compressed in rabbits when compared with humans. Therefore, in order to study the pathogenesis and the microglial responses within this compressed developmental time frame, a larger inoculum than would typically be seen in pediatric CNS TB was used to study the progression of the disease. We believe that the timing of the initial insult at a crucial developmental period is important. The younger age of the rabbits and the high inoculum could also explain why *M. tuberculosis* strain H37Rv, which is typically considered less virulent than the Beijing strains, produced robust disease phenotype in the current study. Future experiments with other *M. tuberculosis* strains, such as from the Beijing family, could elucidate the role of bacterial factors in the pathogenesis of TB meningitis in a developing brain. Finally, direct inoculation with *M. tuberculosis* into the subarachnoid space, as utilized in the current study, does not mimic the natural route of aerosol infection with secondary hematogenous infection. However, this allows for greater reproducibility and induces disease with characteristics similar to humans, enabling further investigation of the host immune response.

### Conclusions

In summary, we have established a reproducible and clinically relevant rabbit model that mimics key features of TB meningitis in young children, who are also disproportionately affected by this form of TB. Future studies will focus on elucidating the role of glial activation in TB and its effects on white matter development and neuronal injury, mechanisms of paradoxical reactions to treatments, novel antimicrobial treatment regimens including host-directed therapies (especially those targeting microglia), and non-invasive imaging to follow progression of disease and response to treatment.

## MATERIALS AND METHODS

All protocols were approved by the Johns Hopkins University (JHU) Biosafety, Radiation Safety, and Animal Care and Use Committees according to the National Institutes of Health guide for the care and use of laboratory animals.

### Bacterial strains

All bacterial stocks were obtained from the laboratory of S.K.J. Logarithmically growing or frozen, titrated stocks of *M. tuberculosis* H37Rv were used as described previously (Harper et al., 2012; Ordonez et al., 2015, 2016a). Prior to infection, the bacterial suspension was washed and re-suspended in phosphate buffered saline (PBS).

### Animal infections

**Fig. 7.**
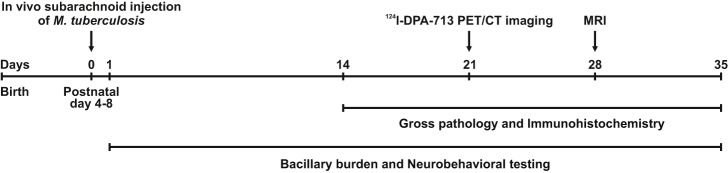
**Schematic of experimental timeline.** *In vivo* subarachnoid injection of *M. tuberculosis* H37Rv at PND 4-8 represents time-point zero. During the experiment, the bacillary burden was quantified and neurobehavioral testing was videotaped and scored. ^124^I-DPA-713 PET/CT imaging occurred at 21 days post-infection and MR imaging at 28 days post-infection.

Freshly prepared *M. tuberculosis* suspension was inoculated into the subarachnoid space of male and female New Zealand White rabbits (Robinson Services Inc., Mocksville, NC) at postnatal day (PND) 4-8. Prior to injection, topical anesthesia (lidocaine; Ferndale IP Inc., Ferndale, MI) was applied and dexmedetomidine hydrochloride (0.2 µg g^−1^; Zoetis, Florham Park, NJ) was provided for sedation. The rabbit was restrained on a board enabling stabilization of the head, and the bregma was palpated and marked. A 28-gauge insulin syringe was used to inject 20 µl of bacterial suspension (over 20 min) into the subarachnoid space via the bregma by trained personnel as previously described (Dai et al., 2010). The bregma was used as the injection site as it is open and easily accessible in young rabbits. Average optical density (OD_600_) of the bacterial suspension was 1.42±0.21. Control (Sham) animals were anesthetized and injected with PBS or were only anesthetized without any injection (Naïve). As we observed no significant differences in CNS pathology or behavior between the PBS-injected (Sham) and non-injected (Naïve) control animals (Fig. S1), we combined both these groups into one uninfected control group in the subsequent experiments. Animals were euthanized with pentobarbital sodium (120 mg kg^−1^) at day 1, day 14, day 21, day 28 and day 35 post-infection and organs were aseptically harvested. The overall study design is presented in Fig. 7.

### Postmortem analysis

Organs from *M. tuberculosis*-infected animals were homogenized and plated onto Middlebrook 7H11 selective plates (Becton Dickinson) to enumerate the bacterial burden using methods described previously (Be et al., 2008; Ordonez et al., 2015). Brains were examined grossly and histologically with specimens fixed with 4% paraformaldehyde and paraffin embedded or placed in cryoprotective solution with 30% sucrose. At least five animals per group were euthanized at indicated time-points. Brain slices were stained with hematoxylin-eosin (H&E) to characterize TB lesions and Ziehl–Neelsen staining for mycobacterium.

### Microglial staining and cell count

Antibody against the microglia/macrophage-specific calcium-binding protein, ionized calcium-binding adapter molecule 1 (Iba-1, 1:500 goat anti-Iba-1, Abcam, Cambridge, MA, ab107159) was used to stain microglia as previously described (Zhang et al., 2015). Every fifth section (thickness of 15 µm) extending caudally from the bregma (site of injection) was stained and examined under a Leica DM2500 microscope (Leica Microsystems Inc., Bannockburn, IL). Four non-overlapping images from the area of the corpus callosum (representative white matter region) in each section were randomly captured at 40× magnification. A total of five sections (20 images) per animal were evaluated. Iba-1-positive cells were counted and the percentage of activated microglia (bushy, round) to total microglial cells [activated and resting (ramified)] was calculated to determine an activation index as previously described (Kannan et al., 2007; Saadani-Makki et al., 2009). Images were analyzed by personnel blinded to experimental groups. Microglial activation was examined 21 days post-infection and data from at least five animals were analyzed for each group.

### Non-invasive imaging

Live *M. tuberculosis*-infected and control animals were imaged within a sealed bio-containment bed (Minerve, Esternay, France) modified in-house to be compliant with biosafety-3 (BSL-3) containment (Davis et al., 2009a,b). Filters (0.22 µm, Whatman) were used at both the inlet and the outlet to contain the bacteria within the device. For MR imaging, animals were imaged inside in-house-designed BSL-3 containment devices with no metallic parts. Rabbits were initially sedated with dexmedetomidine hydrochloride for placement of an intravenous access, and for positioning within the BSL-3 containment device. Anesthesia was then maintained with a mixture of isoflurane (Henry Schein, Melville, NY) and oxygen titrated to effect for the duration of transport and imaging. Animals were injected via the ear vein with 16.24 MBq of ^124^I-DPA-713 for PET/CT or 5 mmol gadolinium-based contrast (Magnevist, Berlex Laboratories, Wayne, NJ) for MR imaging.

#### PET imaging

Radioiodinated DPA-713, a synthetic ligand for TSPO (translocator protein also known as the peripheral benzodiazepine receptor), which is upregulated on mitochondria in activated microglia/macrophages, has been previously validated as a marker of activated macrophages in pulmonary TB (Foss et al., 2013) and was used to monitor microglial activation using methods described previously (Ordonez et al., 2015). Briefly, ^124^I-DPA-713 was synthesized using current Good Manufacturing Practices under a research contract (3D Imaging, Maumelle, AR). Animals were imaged 21 days post-infection using the Mosaic HP PET (Philips, Bothell, WA) and the CT component of the NanoSPECT/CT (Bioscan, Washington, DC) small animal imagers. Images were reconstructed and co-registered using AMIDE 1.0.4 (http://amide.sourceforge.net). Spherical (8 mm^3^ volume) regions of interest (ROI) were drawn around TB lesions visualized on CT and in the same corresponding region (frontal-parietal region) in control animals. Two ROIs were drawn for each animal and standardized uptake values (SUV) were calculated by normalizing ROI activity by correcting for injected dose, animal weight and tracer decay. Data represents four ROIs of *M. tuberculosis*-infected animals and two ROIs from a control animal.

#### MR imaging

Animals were imaged 28 days post-infection using a 9.4T Bruker Avance (Bruker, Billerica, MA) with a Bruker 70 mm diameter volume coil, acquiring multi-slice T_2_-, T_2_*- and T_1_-weighted images. The T_2_-weighted images were acquired using a rapid acquisition with relaxation enhancement (RARE) sequence (rare factor of 8, TR of 5000 ms, effective TE of 32 ms, TE of 8 ms, 2 acquisitions). The T_2_*-weighted images were acquired with a fast low angle shot (FLASH) sequence (TR of 500 ms, TE of 6 ms, and one acquisition).

### Behavioral testing

Animals were videotaped pre- and post-infection and then at least every 7 days for a minimum of 5 min. Behavior and maturation were scored by a blinded observer using a validated scoring system (Chua et al., 2009; Derrick et al., 2004; Kannan et al., 2012; Zhang et al., 2015). A composite behavior score was developed to combine the behavioral and maturation scores with the development of motor deficits as the disease progressed and was based on the following criteria: cliff avoidance, body position, head position, movement direction, eye opening, balance, hop, limb drag, activity duration, activity intensity and seizure activity (Fig. 6A). As the rabbits matured, the composite behavior score increased to its peak. However, as *M. tuberculosis*-infected animals developed signs of infection, the score decreased again, reflecting a regression of milestones and development of deficits. Behavioral testing ended when animals met criteria for euthanasia per our animal protocol. Data from at least 18 animals are presented for each group (30 *M. tuberculosis*-infected and 18 control animals).

### Statistical analysis

Microglial activation and ^124^I-DPA-713 PET/CT SUV data were analyzed using *t*-tests (GraphPad Software Inc., La Jolla, CA). Linear mixed models that accounted for variation among repeated measurements over time within subjects were used to estimate the differences between neurobehavioral scores across time and treatment. The analyses were performed using R version 3.2.2 (R Foundation for Statistical Computing, Vienna, Austria). Where appropriate, the Bonferroni correction for multiple testing was applied. Data are expressed as means±s.e.m. There was no statistically significant difference in behavior or microglial quantification between PBS-injected (Sham) or non-injected control (Naïve) animals so these groups were combined into one control group for statistical analysis (Fig. S1). Bacterial burden is presented as mean±standard deviation (s.d.) on a logarithmic scale as log_10_ colony forming units (CFU). All other data are presented on a linear scale. *P*≤0.05 was considered statistically significant.
